# Ongoing expansion of the worldwide invader *Didemnum vexillum* (Ascidiacea) in the Mediterranean Sea: high plasticity of its biological cycle promotes establishment in warm waters

**DOI:** 10.1007/s10530-015-0861-z

**Published:** 2015-03-04

**Authors:** V. Ordóñez, M. Pascual, M. Fernández-Tejedor, M. C. Pineda, D. Tagliapietra, X. Turon

**Affiliations:** 1 Department of Genetics and IRBio, University of Barcelona, Barcelona, Spain; 2 Institute of Agriculture and Food Research and Technology (IRTA), S. Carles de la Ràpita, Spain; 3 Australian Institute of Marine Science (AIMS), Townsville, Australia; 4 CNR - National Research Council of Italy, ISMAR - Marine Sciences Institute, Arsenale - Tesa 104, Castello 2737/F, 30122 Venice, Italy; 5 Center for Advanced Studies of Blanes (CEAB-CSIC), Acces Cala S Francesc 14, 17300 Blanes, Girona Spain

**Keywords:** Ascidian, Maturity index, Growth rate, Aquaculture, *COI*, Introduced species

## Abstract

Non-indigenous ascidians are of particular concern to aquaculture industry and, paradoxically, the activities associated with it represent an important way to translocate these species worldwide. In 2012 a non-indigenous ascidian was found covering the oyster crops in the Ebro Delta (Western Mediterranean). We have identified the ascidian genetically and morphologically as *Didemnum vexillum* Kott, 2002. This finding indicates that the species is currently expanding its distribution in the Mediterranean Sea, as it has recently been found in the eastern basin (Venice, Adriatic Sea). Introduced populations of *D. vexillum* are found in temperate and cold waters worldwide, and a successful establishment in the Mediterranean implies a remarkable capacity of adaptability to warm, subtropical conditions. We assessed the life cycle (growth and reproduction) of the ascidian at the studied site. The species has a marked seasonal cycle, with regression in the warmest months and reappearance during winter. In spring *D. vexillum* reaches its maximum abundance, followed by a peak in reproduction just before regression. This cycle is reversed with respect to the one observed in colder waters, highlighting a plastic biological cycle of this invader and an hitherto unknown ability to establish itself in warm waters. We also analysed the genetic structure of the population of the Ebro Delta and the one established in the Lagoon of Venice using *COI* sequence data. The low genetic diversity in our samples (three haplotypes) was consistent with what is observed in the introduced populations worldwide. It is likely that the ascidian was introduced with oyster stock from bivalve cultures in the Atlantic French coasts, where the same three haplotypes have been reported. The high boating activity in the Ebro Delta makes further human-mediated transport of the species highly likely, and nearby fishing grounds can be severely affected if invaded. It is urgent to implement measures to prevent the continuous expansion of this ascidian pest in the Mediterranean.

**Electronic supplementary material:**

The online version of this article (doi:10.1007/s10530-015-0861-z) contains supplementary material, which is available to authorized users.

## Introduction

Successful biological invasions must overcome a series of barriers before the introduced species can become established (Richardson et al. 2000; Blackburn et al. 2011). Environmental features of the new range of distribution, such as climate matching, are crucial for the success of nonindigenous species (e.g. Ashton et al. 2007; Bomford et al. 2010; Blackburn et al. 2011). In turn, once established, knowledge of biological features of these species in the new environment (f.i., growth and reproduction cycles) is crucial to assess their invasive potential, and to develop efficient management tools (eradication, containment, and preventive programs).

Among marine introduced species, ascidians have become one of the most important groups, with many nonindigenous species spread around the world (Lambert 2007; Locke and Hanson 2011). Non-indigenous ascidians are of particular concern to aquaculture industry and, paradoxically, the activities associated with it represent an important way to translocate these species worldwide (Naylor et al. 2001; Minchin 2007). Shellfish aquaculture industry is commonly affected by introduced ascidians worldwide (Gittenberger 2009; Fitridge et al. 2012; Aldred and Clare 2014), causing economic losses (Coutts and Forrest 2007; Daigle and Herbinger 2009; Adams et al. 2011). Nonindigenous ascidians overgrow bivalves, adding weight, restricting water exchange and nutrients, and decreasing bivalve productivity (Gittenberger 2009; Daigle and Herbinger 2009).

A non-native didemnid species was found in spring 2012 covering oyster crops in aquaculture facilities in the Ebro Delta, Spain (western Mediterranean Sea). The morphological traits of the colonial ascidian matched those of *Didemnum vexillum*, Kott 2002. This ascidian, recently recorded in the eastern Mediterranean Sea by Tagliapietra et al. (2012) at the Lagoon of Venice (Adriatic Sea), is one of the most important pest species among marine invertebrates due to its high invasiveness, dispersal capabilities, and its high capacity to impact benthic communities (see Lambert 2009 and references therein). The ascidian represents an issue of special concern to aquaculture industry, as the species overgrows commercial shellfish smothering them (Coutts and Forrest 2007; Bullard et al. 2007a; Valentine et al. 2007a). Moreover, *D. vexillum* is not confined to enclosed areas on man-made artificial structures or to aquaculture facilities, but also occurs in open coasts on natural substrates altering the local biota (Mercer et al. 2009) and severely affecting important fishing grounds (Bullard et al. 2007a; Valentine et al. 2007b).

*Didemnum vexillum* is a species native to the Northwest Pacific Ocean (Lambert 2009; Stefaniak et al. 2012), and has become a very successful invader in temperate and cold regions worldwide, including New Zealand, both coasts of North America, the Netherlands, the Atlantic coast of France, Great Britain, Ireland and the west coast of the Iberian Peninsula (Lambert 2009; Griffith et al. 2009; El Nagar et al. 2010; Cohen et al. 2011; Stefaniak et al. 2012 and references therein).

We report here the presence of *D. vexillum* for the first time in the Western Mediterranean. This finding points to a worrisome ongoing expansion of the range of *D. vexillum* in a subtropical sea, which represents an expansion of its introduced range of distribution towards warmer areas. We hypothesize that this expansion is fuelled by a marked plasticity of biological features that allows the species to survive and proliferate in a wide range of temperatures. To test this hypothesis, we characterized morphologically and genetically the populations found in the Mediterranean, and we studied the life cycle of the species in the Ebro Delta, by documenting its abundance, growth, and reproduction. The biological cycle was compared with available information from other areas of distribution. Knowledge of the biology of introduced species in the newly colonized areas is a crucial factor, allowing stakeholders to evaluate and take the appropriate management actions to mitigate their damaging effects to the aquaculture industry and their further spread. Some advice is provided in this respect.

## Materials and methods

### Study site

**Fig. 1:**
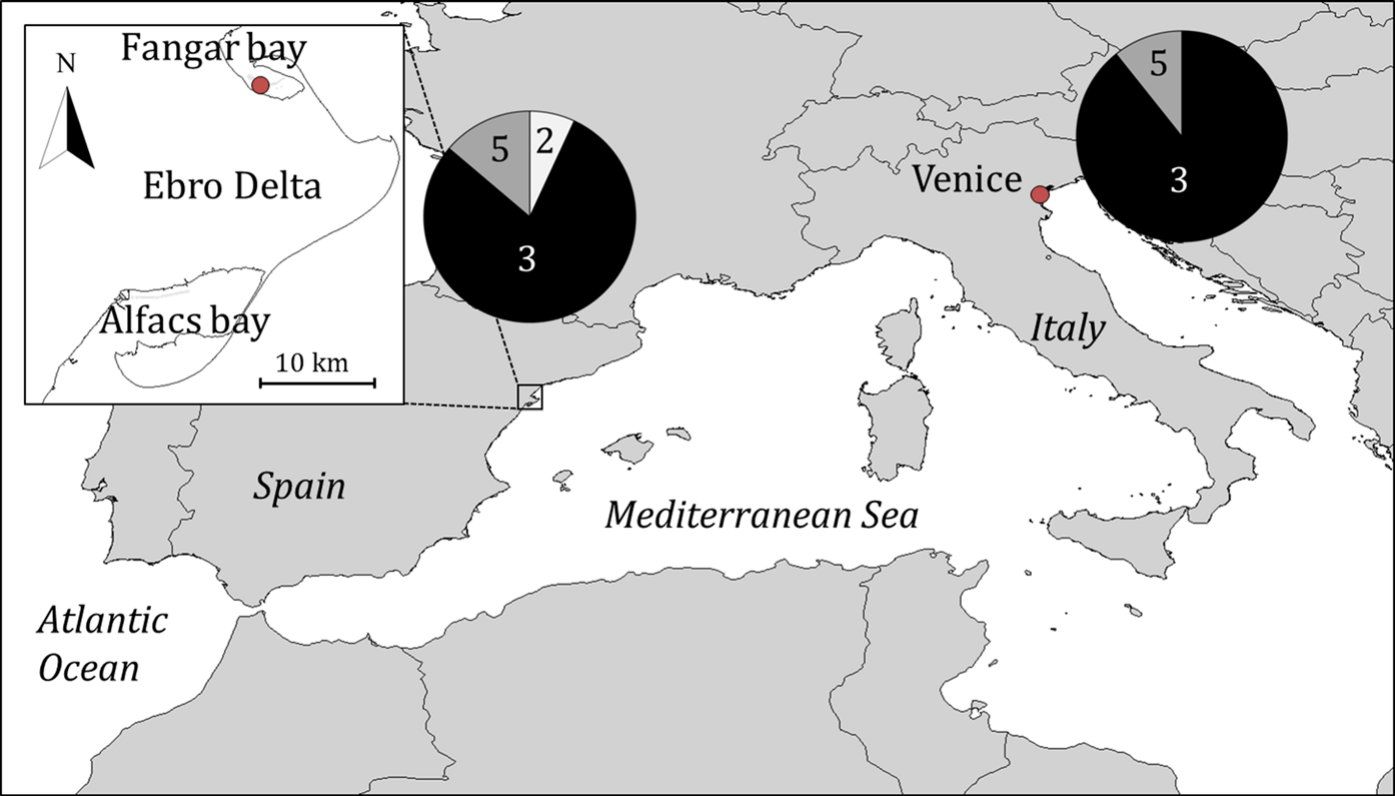
Sampling and monitoring site of *Didemnum vexillum* in Fangar Bay (Ebro Delta, Spain, western Mediterranean Sea), and sampling site in the Lagoon of Venice (Italy, eastern Mediterranean Sea). Haplotype composition (*pie charts*) of both populations is also shown (*numbers* indicate haplotype codes as in Stefaniak et al. 2012)

*Didemnum* *vexillum* was first observed in May 2012 at Fangar Bay, at the northern side of the Ebro Delta (Spain, western Mediterranean Sea, Fig. 1) growing as epifauna on oyster ropes (Supplementary Material, Fig. S1). The species apparently competes for the surface of the oysters’ shell with other organisms, clearly overgrowing algae, polychaetes, ascidians and mussels growing on the oyster ropes (Fig. S1).

Fangar Bay has 9 km^2^ of surface area with a muddy bottom up to 4.2 m depth and houses aquaculture facilities with 77 bivalve rafts. Each raft consists of a rectangular structure of wooden beams arranged in a grid, sustained by cement columns, where the bivalve ropes hang. In a single raft there can be up to 5000 ropes. Both the oyster *Crassostrea gigas* and the mussel *Mytilus galloprovincialis* are grown in the Ebro Delta, but the former is the species most commonly cultured in this bay. *Didemnum vexillum* is most conspicuous on oyster cultures, but it has also been seen growing on the mussel ropes.

Biotic and abiotic parameters of the bay were measured weekly by the staff of the Institute of Agriculture and Food Research and Technology (IRTA) as part of a long term monitoring program. Temperature (°C), salinity (practical salinity scale) and dissolved oxygen percent (%) saturation were measured weekly at 0.5 m water depth using an YSI 556 Handheld Multiparameter Instrument. Water samples were taken at the same depth and were analysed for in vivo chlorophyll *a* using a Turner Trilogy Laboratory Fluorometer.

### Monitoring of abundance and growth cycle

From May 2012 to December 2013 we monitored five ropes with *C. gigas* granted by the owner and placed along separate oyster rafts in the middle of Fangar bay. These ropes had been there since the previous autumn. The ropes were monthly surveyed with the exception of September 2012 and February 2013 when the monitoring could not be done due to logistic problems. In order to obtain an estimate of the covering of *D.* *vexillum*, pictures of each rope were taken with a digital camera from two opposite sides including a ruler to scale every picture. The perimeter of each colony or fragment was manually outlined in every picture with Photoshop CS4. Pictures were subsequently analysed by the Laboratory of Image Analysis and Treatment of the CCiTUB (Scientific and Technological Centre of the University of Barcelona), using the proprietary program IMAT. To obtain a relative estimate of abundance, the total cover of the didemnid on every rope was expressed as area of didemnid per length of rope (in cm^2^/m).

### Reproductive cycle

To study the reproductive cycle we collected on a monthly basis fragments of at least five colonies of the ascidian from June 2012 to December 2013. The fragments were ca. 4 cm^2^ in area and at least 1 cm apart from the margins of the colonies to prevent sampling actively growing tissues for assessing reproductive status. The colonies were taken from different ropes to avoid sampling repeatedly the same clone, as the colonies can reproduce asexually through fission. Samples were fixed in situ in formaldehyde 4 %. Colonies were then dissected under a binocular microscope. Ten zooids were randomly selected per colony in order to determine their reproductive status. As most colonial ascidians, this didemnid is hermaphroditic and protandric, so each zooid was categorized as follows: (1) immature, (2) presence of testis, (3) presence of testis and oocyte, and (4) presence of oocyte alone. We calculated a maturity index per month (MI) as in López-Legentil et al. (2005), averaging the category number of the ten zooids per colony. Further, when there were brooded embryos the whole colony was assigned a status of (5) when they were immature, and (6) when they were well developed larvae with tail and sensory vesicle. Finally, stage 0 was assigned to the month (October 2012) when colonies were absent due to regression. The mean of the five colonies studied per month was then used as a monthly estimate of the MI.

### Statistical analysis

Cross-correlation analyses were carried out to study the relationships of the growth cycle and MI with environmental parameters in the water column: temperature (°C), salinity, levels of oxygen (%) and chlorophyll *a* (µg l^−1^). In cross-correlation analysis, two time series are compared using the Pearson correlation coefficient with an increasing lag of one series with respect to the other. Correlation at time lag 0 is the usual Pearson correlation. Correlations at negative lags relate values in the first series with previous values in the second series. Correlations at positive lags analyse relationships of the values in the first series with future values in the second series.

In order to correct for missing data (September 2012 and February 2013, for both growth cycle and MI), the values of these months were calculated averaging the value of the previous and the posterior months. The package Systat v. 12 was used for these analyses.

### Genetic analyses

DNA sequences were obtained of colonies from the Ebro Delta and from the Venice Lagoon. The latter area was reported as the first occurrence of *D. vexillum* in the Mediterranean Sea (Tagliapietra et al. 2012), but no genetic data were available for this location. Fragments of a total of 29 colonies from different ropes were collected in Fangar Bay (Ebro Delta), and a total of 28 samples from artificial structures in the Arsenale and Lio Grando marinas inside the Lagoon of Venice were also collected with at least 2 m of separation. Samples were fixed in 96 % ethanol in the field and stored at −20 °C in the laboratory. For DNA extraction, we dissected the colonies and pooled ca. 30 thoracic regions of zooids or 10 larvae (when available) from each colony. Total DNA was extracted using QIAamp^®^ DNA Mini Kit (QIAGEN) and DNA for each colony was resuspended in 200 µl of AE buffer.

We used the primers Tun_forward, 5′-TCGACTAATCATAAAGATATTAG-3′ and Tun_reverse2, 5′-AACTTGTATTTAAATTACGATC-3′ described in Stefaniak et al. (2009, 2012), for the amplification of a fragment of the mitochondrial gene *Cytochrome C Oxidase I* (*COI*). PCR amplifications were carried out in a total volume of 20 µl, including 1 × buffer (GoTaq, Promega), 25 nmol MgCl_2_, 0.5 nmol of each dNTP, 4 pmol of each primer, 1U *Taq* Polymerase (GoTaq, Promega) and 1 µl of DNA. The PCRs started with an initial denaturation at 94 °C for 5 min, followed by 35 cycles of a denaturation step at 94 °C for 1 min, an annealing step at 50 °C for 1 min and an elongation step at 72 °C for 1 min 30 s. A final elongation at 72 °C was performed for 7 min. The amplified DNA was purified with Exo-SAP and sequenced (both strands) by Macrogen Inc. Sequences were visually inspected, edited and aligned with BioEdit Sequence Alignment Editor (Hall 1999) to obtain a 586 bp sequence length after trimming. To match previously identified haplotypes, the sequences were aligned with known haplotypes of *D. vexillum* available from GenBank (Stefaniak et al. 2009, 2012).

## Results

### Morphological observation

The colonies grew as encrusting sheets, of yellow-orange colour (Fig. S1). When all the available substrate is occupied they start forming lobes and digitations. The colony and zooid morphology match previous descriptions of the species, and a summary with images is provided in Appendix S1 and Fig. S2 of the Supplementary Material.

### Growth and reproduction

**Fig. 2:**
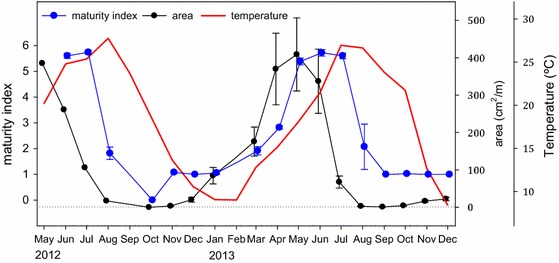
Growth cycle and time-course of the maturity index of *Didemnum vexillum* from May 2012 to December 2013. Monthly area values represent the mean coverage (cm^2^/m ± SE) of the five ropes surveyed (from May 2012 to August 2012 only the value corresponding to the single rope with the ascidian is shown, see text). Maturity index as defined in text, note that a value of 0 was assigned to the month when no colonies were present (October 2012). *Bars* are standard errors. Temperature recordings during this period are also plotted for comparison. *Horizontal dotted line* marks the value of 0 for area

The time course of environmental variables (temperature, chlorophyll *a*, salinity, and levels of O_2_) over the study period is shown in Fig. S3 as monthly means. During the 20 months of monitoring, there was a marked seasonal fluctuation in the coverage of *D. vexillum* in Fangar Bay with a maximum in spring (Fig. 2). From May 2012 to August 2012 only one of the five ropes surveyed was colonized by *D. vexillum.* During summer 2012, the colonies entered in regression and disappeared completely in October 2012. Unfortunately, we do not have data from September 2012, and we cannot tell when exactly *D. vexillum* disappeared from the rope, but it is likely that the ascidian was already missing in that month given the very low cover observed in August 2012. In November 2012 small colonies of *D. vexillum* began to grow on the five ropes, with a mean coverage of 3.33 cm^2^/m. The ascidian grew all along winter, reaching its maximum abundance in spring (May 2013), when they occupied ca. 400 cm^2^/m in average. The colonies of *D. vexillum* started again to regress and, although this time the ascidian did not disappear completely, by September 2013 their coverage was negligible (mean of 0.89 cm^2^/m). By October 2013 colonies of *D. vexillum* started to grow again in all ropes until the last month of our monitoring (December).

**Fig. 3:**
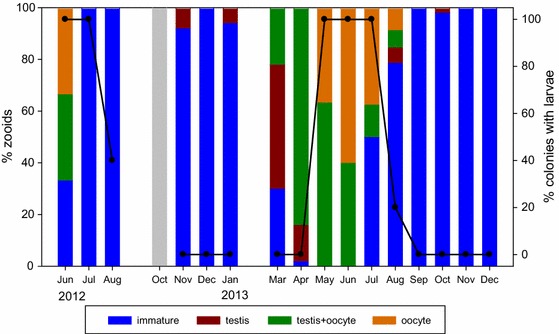
Reproductive status of the zooids in colonies of *Didemnum vexillum* from Fangar Bay (Ebro Delta) over the monitored period. *Columns* indicate the percentage of zooids belonging to each stage (average of five colonies per month). The *solid line* represents the percentage of colonies that were brooding embryos and/or larvae in the basal tunic. *Light grey* column indicates that no colonies were present in October 2012. Sampling could not be carried out in September 2012 and February 2013

As in the growth cycle, there was a strong seasonal fluctuation in the reproductive status of *D. vexillum* zooids (Fig. 3). Immature zooids were dominant in the colonies from July to August onwards in the 2 years surveyed. Unfortunately we do not have data for February 2013 due to logistic problems, but in March 2013 the majority of zooids had already developed gonads, with testis or testis and oocyte. Zooids with only oocytes (i.e., testes already regressed) appeared in May 2013 and were dominant in June 2013 (they were present, but not dominant, in June 2012). Embryos and larvae were present only in late spring and early summer, and in most cases mature larvae and embryos co-occurred in a single colony, although the former were usually dominant. There were still some larvae in incubation in August of both years, when colonies were regressing and almost all zooids were immature.

The MI peaked in June and July 2012, and decreased markedly in August 2012 (Fig. 2). In autumn 2012 and early winter 2013, the ascidians remained immature, but in spring 2013 the MI increased reaching again its maximal levels in late spring-early summer 2013. In August 2013 the MI decreased again. Overall, a clear pattern is evident: growth peaked first, followed by the peak in reproduction, before the highest temperatures were reached (Fig. 2). Interestingly, growth restarted in winter well before water started warming again.

**Fig. 4:**
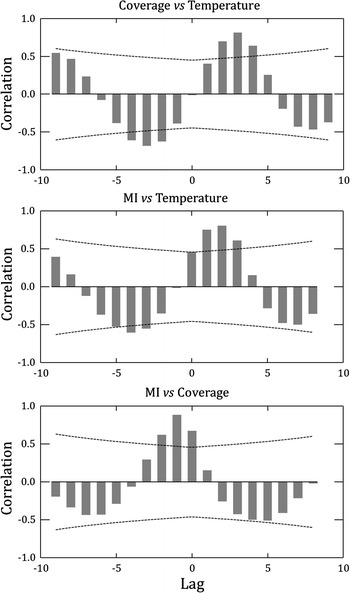
Cross-correlation analyses between the mean monthly coverage (cm^2^/m) and the Maturity index (MI) of *Didemnum vexillum* with temperature (°C). Cross-correlation between MI and coverage is also provided. Data series were lagged with respect to one another and the Pearson correlation coefficient computed for each time lag. The *curved lines* represent the threshold for significant (*p* = 0.05) correlation values. Time lags are in months

Cross-correlation analyses showed a statistical outcome consistent with this general picture. Cover has a positive and significant correlation with the temperatures at time lags of +2, +3 and +4 months (Fig. 4), while MI was significantly related with current temperature (lag 0), but more strongly so with the temperatures of the three following months (time lags +1, +2 and +3). These outcomes are in agreement with the sequential maxima observed of coverage, MI, and temperature (Fig. 2). Conversely, there is a significant negative relationship of the temperature in the 2–4 previous months (lag of −2, −3, and −4) with the coverage of the current month (Fig. 4), and the MI was negatively correlated with the temperature of the 3–4 previous months (time lags −3 and −4). Thus, the periods of high growth and reproductive activity followed sequentially some months after the periods of falling temperatures. On the other hand, cross-correlations between MI and coverage were significant and positive at time lags 0, −1 and −2, and particularly strong at time lag −1 (Fig. 4), which reflects that the cycle of reproduction is delayed with respect to the growth cycle (Fig. 2).

As for other environmental factors, levels of chlorophyll *a* of the two previous months were significantly and negatively correlated with the cover of *D. vexillum* (Fig. S4). Salinity showed only a negative significant correlation of the fifth previous month (lag −5) and there was a positive significant correlation of coverage with the levels of O_2_ at time lag +1 (i.e., with oxygen levels the following month). For the MI (Fig. S5), there was a significant positive correlation with future values of chlorophyll *a* (time lags +2 and +3), and a negative one with values the second previous month (time lag −2). Cross-correlations between MI and salinity were not significant, and with levels of O_2_ they were so only with current levels (time lag 0). Thus, the presence in the water of abundant potential food (phytoplankton as measured by chlorophyll *a* levels, which is higher in summer and early autumn, Fig. S3) did not seem to result in increased growth of the colonies or to trigger reproduction altogether. Salinity and oxygen levels, on the other hand, did not show clear seasonal cycles (Fig. S3), and the few correlations observed with cover and reproduction of the ascidian are difficult to interpret ecologically and may be due to random outcomes or to the effect of other co-occurring factors.

### Genetic analyses

Our *COI* sequences from Fangar Bay (Ebro Delta, Spain) and from the Lagoon of Venice (Italy) matched known sequences of *D. vexillum* from Stefaniak et al. (2009, 2012). Three haplotypes (haplotype numbers as in Stefaniak et al. 2012) were found in Fangar Bay: Haplotype 2 (7 %); Haplotype 3 (79 %) and Haplotype 5 (14 %), and only two in the Lagoon of Venice: Haplotype 3 (89 %) and Haplotype 5 (11 %) (Fig. 1). Haplotype diversities were 0.36 and 0.12, respectively, in Fangar Bay and Venice Lagoon.

## Discussion

Both morphological and genetic data confirmed that the colonial ascidian that grows in Fangar Bay is *D. vexillum* Kott, 2002. The species overgrows cultured oysters and other epifaunal organisms on them. This colonial ascidian is a successful invader of temperate areas (Bullard et al. 2007a; Lambert 2009), and its presence in the Mediterranean represents an expansion of its introduced range and reflects the potential of the species to adapt to warmer, subtropical waters. This potential is further confirmed by the recent finding of this species in the waters of Ecuador (F. Brown, pers. comm.).

Laboratory experiments have shown that *D. vexillum* grows better at the range of ca. 16–20 °C (McCarthy et al. 2007). In temperate waters with cold winters the species experiences regression in winter (e.g., NE America: Bullard et al. 2007a; Valentine et al. 2007a; N Europe: Gittenberger 2007). In Europe, its optimal growing temperatures seemed to be between 14 and 18 °C, with decreases at warmer and colder temperatures and massive die-off at temperatures below 5 °C (Gittenberger 2007). In our study site, with water temperatures in the range of 8–28 °C, the species is close to its warmest limit of thermal tolerance (Zerebecki and Sorte 2011), and it has a seasonal cycle reversed from that in temperate areas, with active growth in winter and regression in summer. Area reduction begins when temperatures exceed 18 °C, and the species regresses completely (as in 2012) or almost so (as in 2013) by the end of summer. Active growth occurs again when water temperature cools below 14 °C, likely from inconspicuous resistance fragments or buds that survive aestivation. In the other Mediterranean locality where the species is present, the Venice Lagoon in the Adriatic Sea, with more extreme temperatures than the Ebro Delta (from 0 to >30 °C surface temperature), growth peaks also in spring (DT, pers. obs.), but the species passes through two periods of arrested growth, one in winter and one in summer (Tagliapietra et al. 2012).

The peak of cover values in *D. vexillum* occurs 1–2 months before the peak in MI, which suggests that this species has some degree of resource partitioning between growth and reproduction. Such a trade-off has also been found in other colonial ascidians (López-Legentil et al. 2005; Pérez-Portela et al. 2007; López-Legentil et al. 2013). There is little information about the reproductive cycle of *D. vexillum*, but in New Zealand (temperature range ca. 9–23 °C) there are embryos in the basal tunic all year round, with minima in winter (Fletcher et al. 2013a). Recruitment occurs from summer to early autumn in temperate waters (e.g., NE America: Osman and Whitlatch 2007; Valentine et al. 2009; Bullard et al. 2013; NW America: Sorte and Stachowicz 2011). In New England, recruitment in coastal sites occurred over 3.5–5 months in the range of 14–20 °C and ceased at 9–11 °C, with local climatic conditions adjusting the recruitment period (Valentine et al. 2009). In New Zealand, recruitment occurs over a longer period of time, from late spring to early winter, and is interrupted during the austral winter (Fletcher et al. 2013a). In the other Mediterranean locality (Venice Lagoon), Tagliapietra et al. (2012) indicated the presence of larvae in the colonies in June. In our case, we didn’t assess recruitment, but mature larvae were present in all colonies in May–July, with a range of temperatures of 18–27 °C. Larval release is likely to be concentrated at the end of the developmental period in July-early August.

*Didemnum vexillum*, therefore, seems to have a highly plastic life-cycle, finely tuned to thermal conditions, with winter regression in colder environments and summer regression in warmer environments such as the Mediterranean. Life-history plasticity is common in ascidians (Millar 1970), and there are other instances of colonial ascidians that hibernate in the North Atlantic and aestivate in the Mediterranean (e.g., Mukai 1977; De Caralt et al. 2002). Summer is in general the unfavourable season in the Mediterranean for many invertebrates (Coma et al. 2000; Coma and Ribes 2003), and colonial ascidians in particular (e.g. Turon 1988; Turon and Becerro 1992; López-Legentil et al. 2013).

Even though Fangar Bay is a shallow embayment with freshwater inputs, the levels of salinity did not fluctuate widely during the study period and the same can be said for the oxygen levels. It is not surprising, therefore, that no meaningful correlation with these parameters could be found in our study. For chlorophyll *a* content, on the other hand, a negative relationship of coverage and MI with the chlorophyll *a* values of the previous months was found, which seems paradoxical. However, we attribute this outcome to the seasonal pattern in the abundance of the phytoplankton, highly correlated with the temperature of the water (Spearman rank correlation *R* = 0.791, *p* < 0.001), rather than to an intrinsic negative influence of the levels of chlorophyll *a* (and hence phytoplankton abundance) in the growth and reproduction of the species.

Multiple vectors of introduction of *D. vexillum* have been identified (Lambert 2009; Herborg et al. 2009). The natural dispersal capabilities of the species are restricted to local scales (meters to kilometres, Fletcher et al. 2013b), so anthropogenic transport is assumed for long distance movements. Ballast water is unlikely to be a relevant vector (Herborg et al. 2009) due to the very short larval phase of the species, while hull-fouling in fast-moving vessels may be improbable due to low attachment strength (Murray et al. 2012). On the other hand, transport as fouling on slow-moving vessels (barges or recreational boats) and as epifauna on aquaculture stock (Dijkstra et al. 2007; Denny 2008) are likely to be important vectors of introduction (Herborg et al. 2009). In our case, as there was no long-distance ship traffic in the concerned bays or nearby areas, transfer via aquaculture activities seems the most likely vector. European regulations of oyster movements concern mostly food safety procedures, with no specific controls on accompanying species. New scope for action is given by European regulation 1143/2014 on the prevention and management of alien species, still to be implemented. We strongly advocate the inclusion of *D. vexillum* on the UE species list being compiled as an instrument that will allow further control actions.

The populations analysed showed a low genetic diversity (three haplotypes in the Ebro Delta and two in Venice Lagoon). However, this is true of most introduced populations of *D. vexillum* worldwide. In fact, only six haplotypes had been previously found in introduced populations of the species, compared to 22 in the putatively native region in NW Pacific (Stefaniak et al. 2012). The three haplotypes found in the Mediterranean are present in the native zone. Furthermore, haplotype 3 was present in all introduced areas, while the other haplotypes had been found previously in Europe and NW America (haplotype 2) and in Europe, NE America and New Zealand (haplotype 5). In the Ebro Delta, according to fishermen, the oyster spat came from the Atlantic coast of France (mostly from Arcachon and La Rochelle areas). The few sequences of *D. vexillum* available from the potential source localities (Stefaniak et al. 2012) comprised three specimens from Arcachon and three from La Rochelle, having the same three haplotypes found in the Mediterranean. Indeed, all French Atlantic samples (23 specimens) in Stefaniak et al. (2012) had these same three haplotypes, strongly suggesting that the ascidian was introduced as epifauna with the oyster spat from this area. Nevertheless the low number of individuals analysed in French populations and the low polymorphism of the marker used makes it difficult to assess genetically the source of the founders and further analyses should be undertaken to settle this issue.

Our results show that *D. vexillum* is already widely distributed in the Mediterranean, as it has been found in both the eastern and western basins. In the Ebro Delta, according to local farmers, *D. vexillum* has been present for at least 10 years, although it never reached the abundance levels observed in the last 3 years. This species creates an unwanted fouling that diminishes the visual appeal of the oysters, and cleaning oysters before selling them is time-consuming and costly. Although at its present stage of development the ascidian apparently does not smother the oysters, it has been reported elsewhere that covering by *D. vexillum* affects bivalve growth (Fletcher et al. 2013c). The species seems to be proliferating, as we detected a higher abundance in 2013 than in 2012 (authors pers. obs.). Also, some colonies have been seen in 2012 and 2013 in the southern bay of the Ebro Delta (Alfacs Bay, authors pers. obs.), another site with aquaculture (mussels and oysters) facilities, which suggests that the species is colonizing nearby localities.

The finding of the ascidian in increasing abundances in an area of important aquaculture production and with nearby fishing grounds should raise concern, as *D. vexillum* is capable not only of causing damage to bivalve farms, but also to proliferate in deeper waters including trawling bottoms (Bullard et al. 2007a; Valentine et al. 2007b; Mercer et al. 2009). While the summer regression and the scarcity of hard substrate in the Ebro Delta may have contributed to limit its expansion so far, the species has biotic features that can boost its proliferation. In addition to plastic life-cycles, it has a high rate of inter-colony fusion in introduced populations (Smith et al. 2012) and a capacity of asexual reproduction through fragmentation and reattachment (McCarthy et al. 2007; Bullard et al. 2007b; Valentine et al. 2007a; Morris and Carman 2012). The Ebro Delta bays are enclosed areas with limited connection to the outside, which restricts potential dispersal of the ascidian to the adjacent areas by natural means. On the other hand, slow-moving vessels are commonly used for sailing through the aquaculture facilities and between bays, thus representing a potential factor of man-mediated dispersion of the ascidian all along the Ebro Delta and adjacent coasts.

The fact that the species seems so far restricted to embayments may help setting up contingency measures within these internal borders (sensu Forrest et al. 2009). In New Zealand, local scale eradication of *D. vexillum* was attempted at Shakespeare Bay without success (Coutts and Forrest 2007). However, a sustained (2006–2008) intensive surveillance and eradication program was set up afterwards and obtained a substantial reduction or eradication of populations in the Marlborough Sounds area, together with an almost complete lack of vessel infestation (and thus potential for further spread) (Forrest and Hopkins 2013). Eradication attempts in Holyhead Marina (Wales, UK) were less successful and highlighted the crucial importance of knowledge of the reproductive periods (Sambrook et al. 2014). Moreover, prevention measures can be deployed to clean infested bivalve stock in order to avoid further introductions via aquaculture transfers, but the methods assayed are time-consuming and not free of environmental concerns (Denny 2008) and can even decrease the survival of the stock (Switzer et al. 2011). At present, then, we suggest that surveillance of boats operating in the farms (particularly those travelling to aquaculture settings in other bays) and of oyster stock is the best strategy to prevent further spread of this pest. Unfortunately, there is no specific regulation concerning compulsory cleaning or monitoring so far and it is desirable that these measures are implemented and enforced.

We conclude that we may well be in the verge of a potentially important ecological and economic issue given the capacity of *D. vexillum* to thrive in warmer environments than those previously reported. The biological knowledge gained here may assist to counteract the impact and spread of the species. For instance, seeding new oyster stock during the months when larvae are present in the water column can be avoided. In addition, harvesting can be concentrated during the period of regression of the colonies to avoid cumbersome cleaning of shells and generation of potentially viable fragments. Close monitoring of the Ebro Delta aquaculture facilities and nearby coasts is necessary in the forthcoming years, and stringent control measures should be implemented in the short term if the species proliferation continues.

## Electronic supplementary material

Below is the link to the electronic supplementary material.

Supplementary material 1 (DOC 2737 kb)
