# Supplementary material for: Ongoing expansion of the worldwide invader Didemnum vexillum (Ascidiacea) in the Mediterranean Sea: high plasticity of its biological cycle promotes establishment in warm waters
Source: Biol Invasions. 2015 Mar 4;17(7):2075–85. doi: 10.1007/s10530-015-0861-z (PMC4513794; doi:10.1007/s10530-015-0861-z)
Supplement: Supplementary file 1 — Supplementary material 1 (DOC 2737 kb) [file 10530_2015_861_MOESM1_ESM.doc]

**ELECTRONIC SUPPLEMENTARY MATERIAL**

**Biological Invasions**

**Ongoing expansion of *Didemnum vexillum* in the Mediterranean Sea: biological cycle and genetic structure of a worldwide invader**

Ordóñez V, Pascual M, Fernández-Tejedor M, Pineda MC, Tagliapietra D, Turon X

Corresponding author: X Turon, Center for Advanced Studies of Blanes (CEAB-CSIC), Acces Cala S Francesc 14, 17300 Blanes (Girona), Spain, e-mail: [xturon@ceab.csic.es](mailto:xturon@ceab.csic.es)


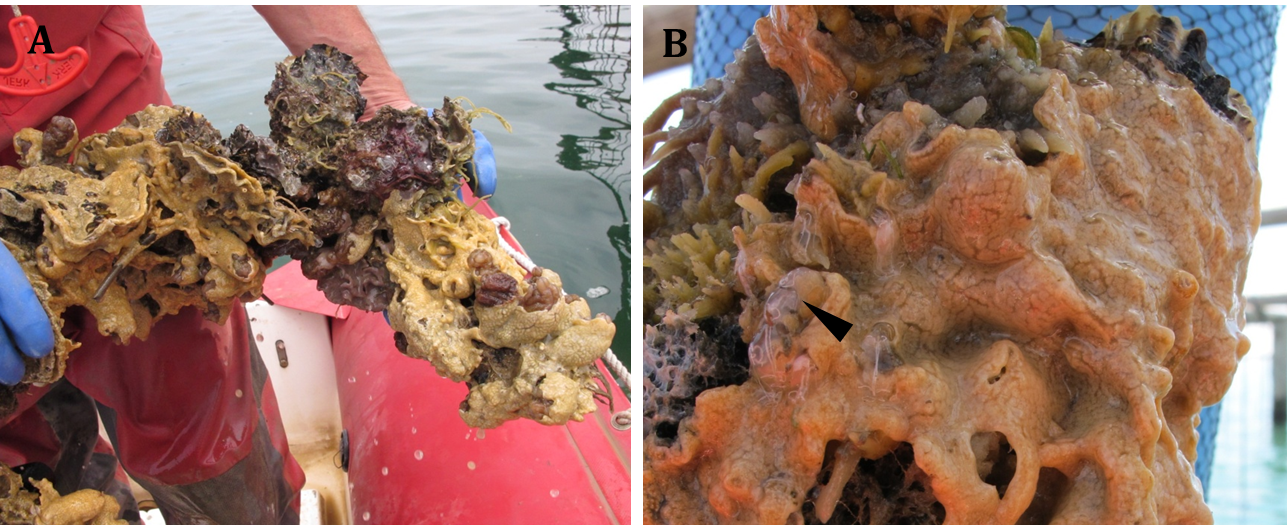


**Figure S1** (A) Colonies of *Didemnum* *vexillum* in oyster ropes at Fangar Bay (Ebro Delta, Spain) overgrowing oysters and their epifauna and (B) close-up view of the ascidian overgrowing other ascidians such as *Clavelina lepadiformis* (arrowhead).

**Appendix S1.** Morphological characters

The zooids are arranged in systems, separated by darker meandering zones with less dense spiculation. The spicules in the tunic are not densely distributed. They are mostly between 20 and 30 µm in diameter, with relatively short and pointed rays, about 8-9 in optical section (Fig. S2A). The zooid morphology is typical of the genus. The thorax has a wide atrial aperture without languet, thoracic lateral organs posteriorly situated and a retractor muscle originating in the oesophageal neck region. When mature, the zooids have a spherical testicle surrounded by a sperm duct with 8-9 coils and one developing oocyte. Larvae (Fig. S2B) are incubated in the basal tunic of the colonies. They are about 500 µm long (in the coiled stage), and feature 3 adhesive papillae and 6 pairs of elongated ectodermal ampullae (Fig S2C).


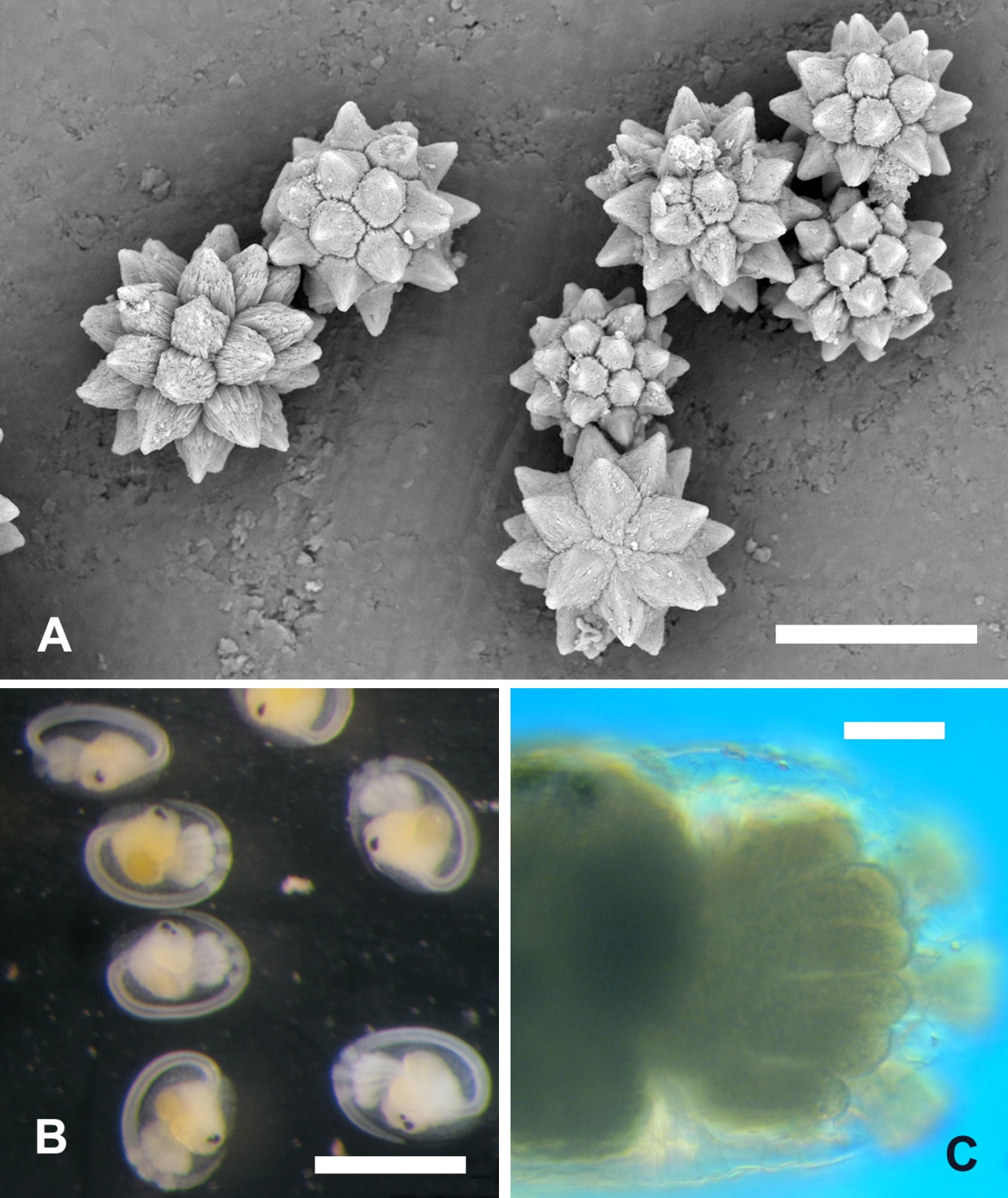


**Figure S2** (A) Image of the spicules in the tunic of colonies of *Didemnum vexillum* from the Ebro Delta. (B) View of unhatched larvae obtained from the basal tunic. (C) close-up of the anterior part of a larva, showing ectodermal ampullae. Scale bars: A, 25 µm; B, 500 µm; C, 50 µm

**Figure S3.** Abiotic parameters (temperature, salinity, levels of O_2_), and levels of chlorophyll *a* (Chl *a*) in the Fangar bay (Ebro Delta, Spain) during the monitoring period (monthly means).


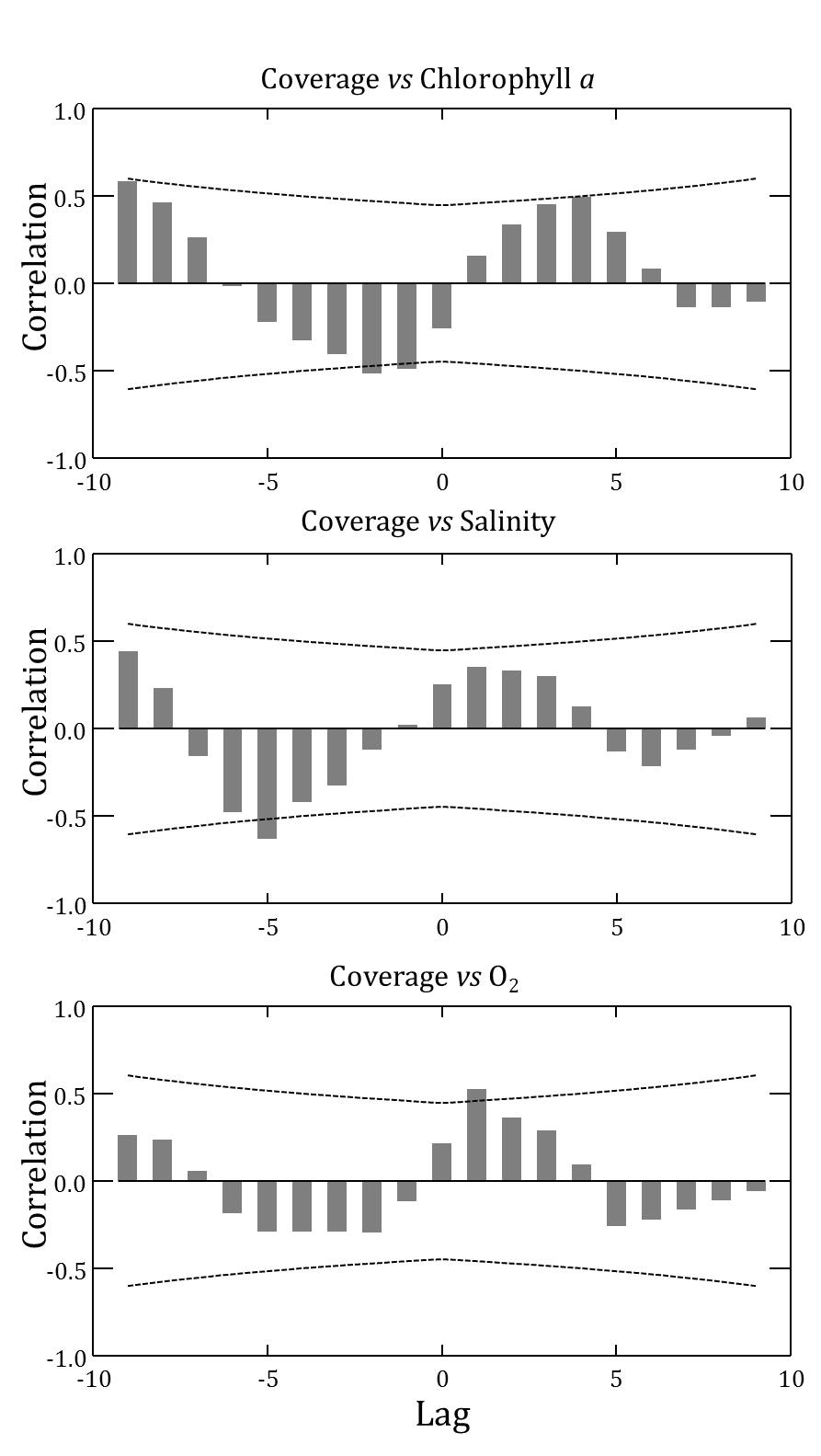


**Figure S4.** Cross-correlation analyses between the mean monthly coverage (cm^2^/m) of *Didemnum vexillum* and chlorophyll *a* (µgL^-1^), salinity, and levels of O_2_ (%) in the water column. The curved lines represent the threshold for significant correlation values (p = 0.05). Time lags are in months.


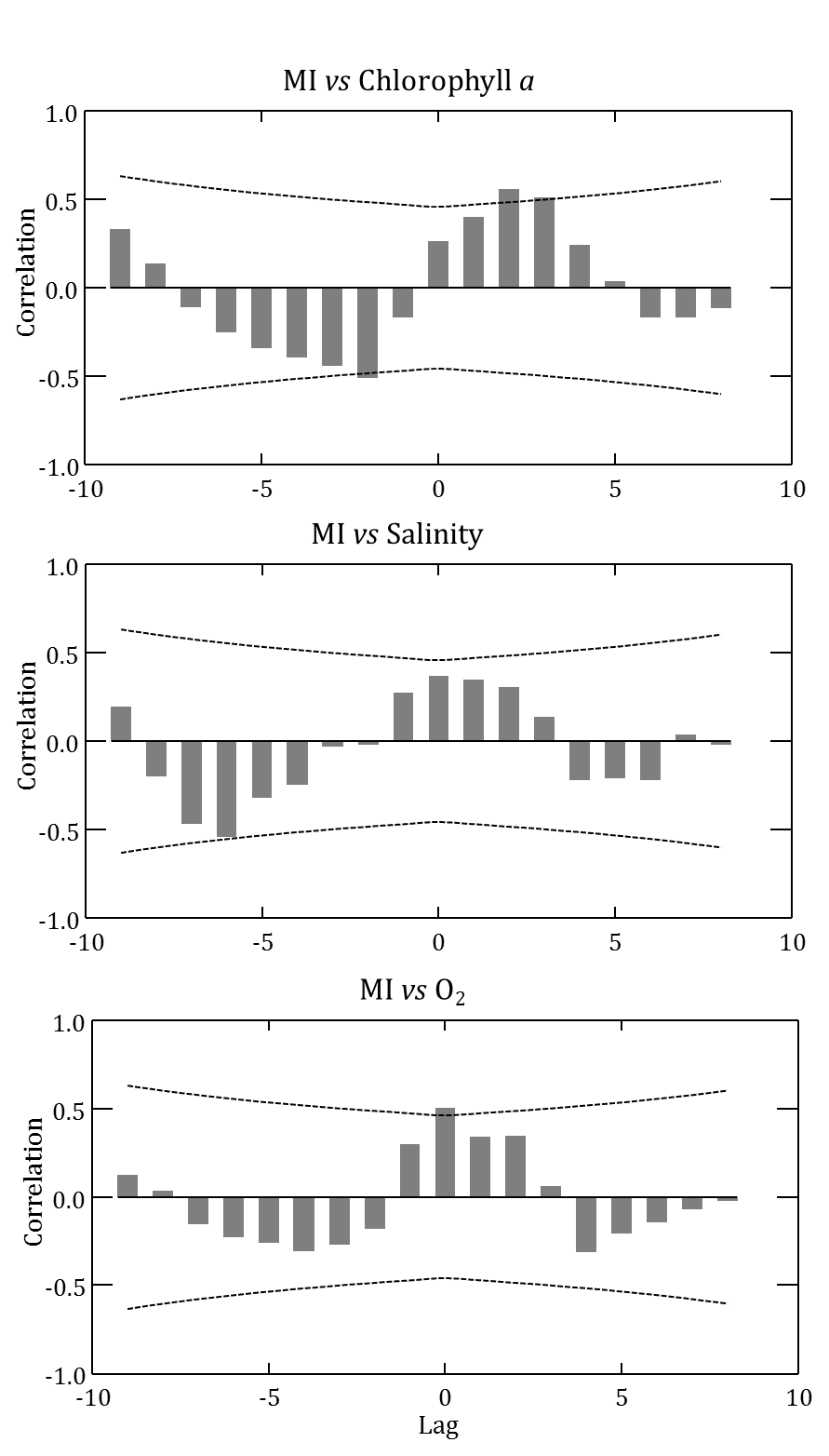


**Figure S5.** Cross-correlation analyses between the Maturity Index (MI) of *Didemnum vexillum* with chlorophyll *a* (µgL^-1^), salinity, and levels of O_2_ (%) in the water column. The curved lines represent the threshold for significant (p = 0.05) correlation values. Time lags are in months.
